# Behavior of Ni20Cr Alloy in Molten Nitrate Salts

**DOI:** 10.3390/ijms23147895

**Published:** 2022-07-18

**Authors:** Nestor Belisario Gomez-Guzman, Daniel Lopez-Dominguez, Cinthya Dinorah Arrieta-Gonzalez, Jan Mayen, Eduardo Porcayo-Palafox, Jose Guadalupe Chacon-Nava, Jose Gonzalo Gonzalez-Rodriguez, Jesus Porcayo-Calderon, Roberto Ademar Rodriguez-Diaz

**Affiliations:** 1 Centro de Investigación en Ingeniería y Ciencias Aplicadas, Universidad Autónoma del Estado de Morelos, Avenida Universidad 1001, Cuernavaca 62209, Mexico; nbgg@outlook.es (N.B.G.-G.); ing.danielld@gmail.com (D.L.-D.); porcayo@gmail.com (E.P.-P.); ggonzalez@uaem.mx (J.G.G.-R.); 2 Tecnológico Nacional de Mexico-Instituto Tecnológico de Zacatepec, Calzada Instituto Tecnológico 27, Zacatepec 62780, Mexico; cdaglez@gmail.com; 3 CONACYT-CIATEQ A.C., Zona Industrial Eje 126 No. 225, San Luis Potosí 78395, Mexico; jan.mayen@ciateq.mx; 4 Centro de Investigación en Materiales Avanzados (CIMAV), Miguel de Cervantes 120, Complejo Industrial Chihuahua, Chihuahua 31136, Mexico; jose.chacon@cimav.edu.mx; 5 Departamento de Ingenieria Quimica y Metalurgia, Universidad de Sonora, Hermosillo 83000, Mexico; 6 Tecnológico Nacional de Mexico-Tecnológico de Estudios Superiores de Coacalco, Av. 16 de Septiembre 54, Coacalco de Berriozábal 55700, Mexico; rdiaz.unam@gmail.com

**Keywords:** Solar Salt, lanthanum nitrate, corrosivity, Ni20Cr alloy

## Abstract

This study reports the behavior of the Ni20Cr alloy in molten nitrate salts. Its behavior was evaluated in the eutectic mixture called Solar Salt (binary salt) and in a ternary mixture (90% Solar Salt and 10% lanthanum nitrate). The addition of lanthanum nitrate was performed to determine if the presence of the La^3+^ cation could act as a corrosion inhibitor. Through mass loss and potentiodynamic polarization studies, the effects of both electrolytes on the corrosion resistance of the alloy at 300, 400, and 500 °C and at exposure times of 250, 500, 750, and 1000 h were determined. The results showed an increase in the corrosivity of the ternary salt, due to a decrease in its melting point and an increase in the concentration of nitrate ions. However, it was observed that the La^3+^ cations formed a protective layer (La_2_O_3_) on the alloy surface. In both corrosive media, the Ni20Cr alloy showed excellent corrosion resistance, due to its ability to form protective layers of Cr_2_O_3_, NiO, and NiCr_2_O_4_, in addition to the presence of a layer of La_2_O_3_ in the case of the ternary salt.

## 1. Introduction

In countries with a high availability of solar energy resources, their use in generating electricity from concentrated solar power plants (CSP) has been of great interest. The continuous operation of CSP plants has been guaranteed using molten salts that assume the function of thermal energy storage systems [1]. Molten salt acts as a heat transfer fluid (HTF) transferring its thermal energy to the water-steam system used to generate electricity. Although CSP plants have a lower efficiency than conventional plants, their technology has a great advantage; it contributes to the reduction in CO_2_ emissions [1].

However, in recent years, there has been a growing interest in the corrosion problems that occur in CSP plants due to the corrosive effect of HTFs on the materials with which it is in contact. Various studies have been reported in the literature to understand the behavior of materials that are in contact with an HTF [2,3,4,5,6,7,8]. In general, HFTs have a low melting temperature, low viscosity, and low vapor pressure. These properties, considered as advantages in the operation of a CSP plant, are a disadvantage for the structural integrity and chemical stability of the materials. The stability of the materials used for heat exchange and storage is important and essential for the safe and reliable operation of a CSP plant.

Currently, there is a wide range of HFTs (carbonate, fluoride, chloride, and nitrate) used in CSP plants, and those based on nitrates have the lowest melting point and relatively good thermal stability (below 600 °C). The prolonged exposure of materials in this type of environment has caused their degradation by corrosion processes with the various species formed by the fusion and thermal degradation of this type of salt [1,3]. Many of the materials used are Fe-based or Ni-based, and their corrosion resistance depends on the development of a protective oxides layer (Cr_2_O_3_, Al_2_O_3_) and the presence of other alloying elements that mainly improve their mechanical properties [2,3,7,9]. It has been observed that the content of both Ni and Cr improves the corrosion resistance; with a higher content of these elements, the corrosion resistance is increased [2,6,10,11,12]. Therefore, the various studies carried out to date have indicated that nickel-based alloys show excellent performance in various HFT fluids [4,5,7].

Because the Ni20Cr alloy is considered a model system that would represent the expected behavior of Ni-based superalloys [13], its performance in molten nitrate salts is reported in this study. Although various studies have indicated that the Ni20Cr alloy shows a remarkable performance in molten salts rich in chlorides and vanadium species [14,15,16], to date, its performance in molten nitrates has not been reported. Its behavior was evaluated by means of mass change tests, at different temperatures and immersion times, and by means of potentiodynamic polarization curves. As a corrosive medium, the eutectic mixture of sodium nitrate and potassium nitrate called Solar Salt was used. Additionally, this binary mixture (Solar Salt) was modified with the addition of lanthanum nitrate in order to determine if the presence of the cation La modifies the degradation process and is capable of forming a protective layer on the surface of the alloy. 

## 2. Materials and Methods

### 2.1. Test Material

Ni20Cr alloy was used as the test material. The alloy was obtained from the melting, in an induction furnace in quartz crucibles, of the commercial alloy powder Metco 43C. According to the manufacturer’s information, its elemental composition is 80% Ni-20% Cr (% by weight). The elemental chemical composition was verified by the EDS technique, and the composition coincides with that reported by the manufacturer. Specimens with dimensions of 10 × 5 × 3 mm were cut from the obtained ingots, which were used in the corrosion tests. The specimens were roughened with silicon carbide paper from grade 120 to grade 600, and they were later washed with distilled water, ethanol, and acetone and dried in air.

### 2.2. Corrosive Medium

As a corrosive medium, the heat transfer fluid called “Solar Salt” was used, which consists of a mixture of 60% NaNO_3_ and 40% KNO_3_ (% by weight). The mixture was formulated from analytical-grade reagents NaNO_3_ (≥99.9 Sigma-Aldrich, St. Louis, MO, USA) and KNO_3_ (≥99.9%, Golden Bell). Because these types of salts are highly hygroscopic, it was necessary to dry them before mixing (60 °C, 24 h), and they were later mixed and pulverized in a ball mill (Tinius Olsen Model TO-441) to ensure their homogenization. Subsequently, the mixture was stored hermetically and maintained at 60 °C to avoid its hydration until its final use in the corrosion tests. Additionally, a mixture of 90% Solar Salt and 10% La(NO_3_)_3_ (% by weight) was used. For simplicity, this mixture is herein referred to as the “ternary salt”. The main purpose of the ternary salt is to determine whether the presence of La^3+^ cations can act as corrosion inhibitors.

### 2.3. Corrosion Tests

The behavior of the Ni20Cr alloy was determined by gravimetric tests during 250, 500, 750, and 1000 h at different temperatures, 300, 400, and 500 °C. The tests were carried out under static air conditions in alumina crucibles, 20 mL, inside an electric furnace (FB1410M, Thermo Scientific, Waltham, MA, USA). The tests were carried out in triplicate and a fourth specimen was used for subsequent analysis (SEM-EDS, DRX). The specimens were fully packed with sufficient corrosive medium to ensure their complete immersion at the test temperatures.

The specimens removed at the different established times were ultrasonically cleaned with hot water, 60 °C, to eliminate any soluble compound, subsequently dried, and weighed. The process was repeated as many times as necessary until a constant weight was obtained. This weight was the one used to determine the variation in mass of the alloy as a function of time. Based on the initial reaction area of the specimens, as well as their initial and final weight, their mass change was evaluated according to the following expression [6]:

(1) $$\frac{\Delta m}{A_{o}}=\frac{m_{i}-m_{f}}{A_{o}}$$

where the term on the left side of the expression represents the mass per unit area (lost or gained), *m_i_* is the initial mass, *m_f_* is the final mass at time *t*, *A_o_* is the initial reaction area of the sample, and Δ*m* is the mass change experienced by the sample. The mass changes thus determined represent the overlapping effects of mass gain due to corrosion, and the mass loss due to dissolution and detachment of corrosion products.

Additionally, the electrochemical behavior of the Ni20Cr alloy was determined through potentiodynamic polarization curves. The experimental arrangement consisted of a three-electrode cell, where the working electrode was the Ni20Cr alloy, a platinum wire (0.5 mm in diameter) was used as a pseudo-reference electrode, and a platinum mesh was used as a counter electrode. The electrical connection of the samples was made with a 0.5 mm diameter Nichrome (Ni20Cr) wire welded using the spot-welding technique. The wires were insulated using alumina tubes, and the sample-ceramic tube junction was sealed with moldable ceramic cement. The polarization curves were made by polarizing the working electrode from −300 to 1000 mV with respect to its corrosion potential (Ecorr) at a scanning speed of 1 mV/s. Prior to the measurements, the open-circuit potential was measured until a stable value (±5 mV) was obtained; in general, the time to achieve this was 15 min. Measurements were made with a potentiostat-galvanostat (Gill AC, ACM Instruments, Grange-over-Sands, UK).

### 2.4. Complementary Analysis

Surface and cross-sectional analyses of the corroded samples were performed with a scanning electron microscope (SEM, JEOL JSM-IT500, Jeol Ltd., Tokyo, Japan; FE-SEM, S-4800, Hitachi, Tokyo, Japan) equipped with an X-ray energy-dispersive spectrometer (EDS). The surface of the corroded samples was analyzed by X-ray diffraction (XRD, Bruker D8 Discover AXS GmbH, Germany) in the range of 10° ≤ 2θ ≤ 100° with a step 0.003° and a time of 320 s per step.

## 3. Results and Discussion

### 3.1. Corrosion Tests

**Figure 1:**
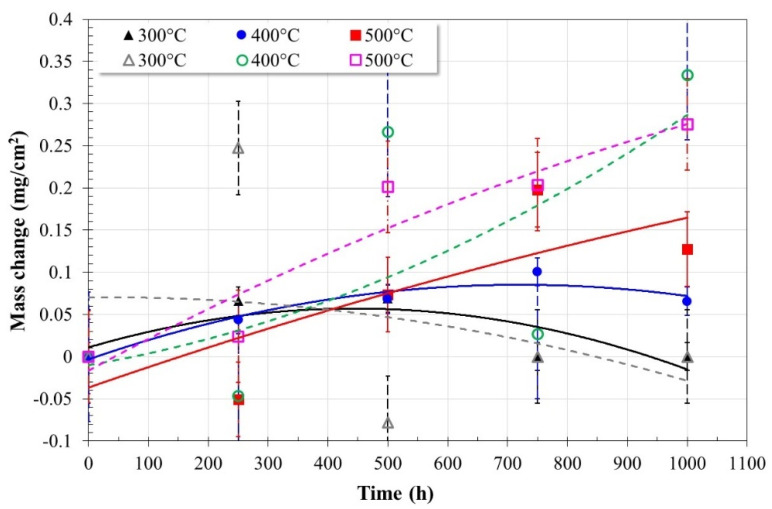
Mass change of Ni20Cr alloy immersed in Solar Salt (continuous lines) and Ternary salt (dotted lines).

Figure 1 shows the variation in mass of the Ni20Cr alloy during the different immersion times in corrosive media at the different test temperatures. The mass changes observed show the ability of the alloy to form stable and strongly adherent protective layers on its surface or its inability to self-heal.

From the graphs in Solar Salt at 300 °C, the Ni20Cr alloy showed a continuous mass gain up to 500 h of immersion and, at longer times, a decrease equivalent to the mass gained initially. The observed trend suggests that strongly adhered corrosion products were initially formed on the surface of the alloy, but detachment or dissolution of these occurred at longer times. However, in addition to this, some authors have suggested that an initial behavior such as the one observed can be attributed to the surface finish rather than the response of the material [7]. At 400 °C, a constant increase in mass gain was observed up to 750 h of immersion and, subsequently, a slight decrease at the end of the test. This suggests that the initial formation of corrosion products strongly adhered to the surface of the alloy and their subsequent detachment or partial dissolution. At 500 °C, a loss of mass and a subsequent gain were initially observed up to 750 h of immersion and, at the end of the test, a subsequent decrease. This indicates that at this temperature, the alloy initially underwent a constant dissolution process and, subsequently, adherent corrosion products formed on its surface.

In general, in Solar Salt, at immersion times greater than 500 h, the effect of temperature on the corrosion process of the alloy was observed, namely, increasing the temperature increased the mass gain, which indicates an increase in the metallic dissolution process and, therefore, a greater presence of corrosion products. This is justifiable given that corrosion in molten nitrates occurs because of their corrosive ions (O^2−^, NO^−2^) formed by the thermal decomposition of nitrate ions (NO^−3^) whose presence is accelerated at temperatures above 500 °C [6,12]. This shows the direct relationship between the metal dissolution process and the ability of the alloy to self-heal with both temperature and immersion time. In addition, although the microstructure is an important factor, at the highest temperature evaluated in this work, no significant effect has been reported that affects its behavior under aggressive conditions [13].

The mass gain detected at the highest test temperature is lower than those reported for other nickel-based alloys [5], and it has also been suggested that nickel-based alloys are passivated in molten nitrate salts [7]. In general, the behavior represents the combination of different processes that occur on its surface, namely, metallic dissolution, growth of oxides, precipitation of corrosion products, and scaling, and its tendency can indicate the kinetics and mechanism of the process of corrosion [12,17].

Based on the above and due to the low changes in mass recorded, it can be inferred that the Ni20Cr alloy shows excellent corrosion resistance in Solar Salt. This is consistent with previously reported studies where the Ni20Cr alloy and its coatings [14,15,16] exhibited excellent corrosion resistance in the presence of more aggressive molten salts (rich in chlorides and vanadates) and at higher temperatures.

**Figure 2:**
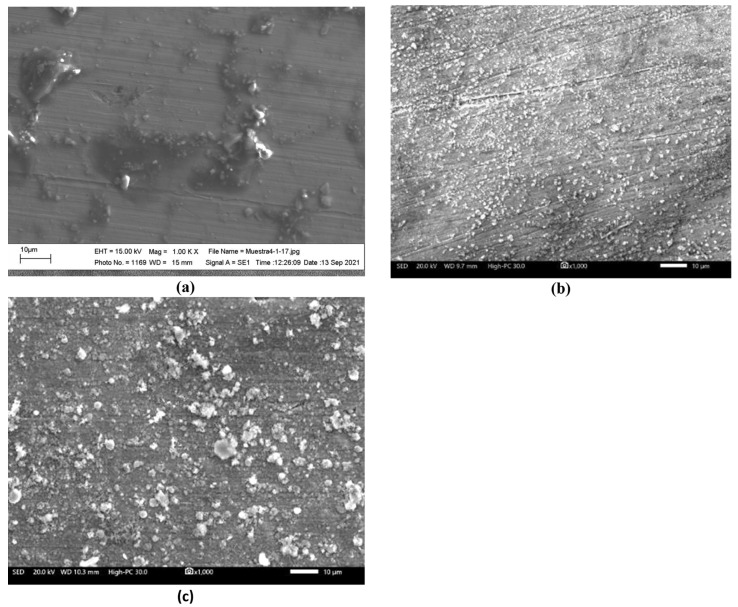
Morphological aspects of the Ni20Cr alloy surface immersed in Solar Salt after 1000 h of immersion and at different test temperatures: (**a**) 300 °C, (**b**) 400 °C, and (**c**) 500 °C.

**Figure 3:**
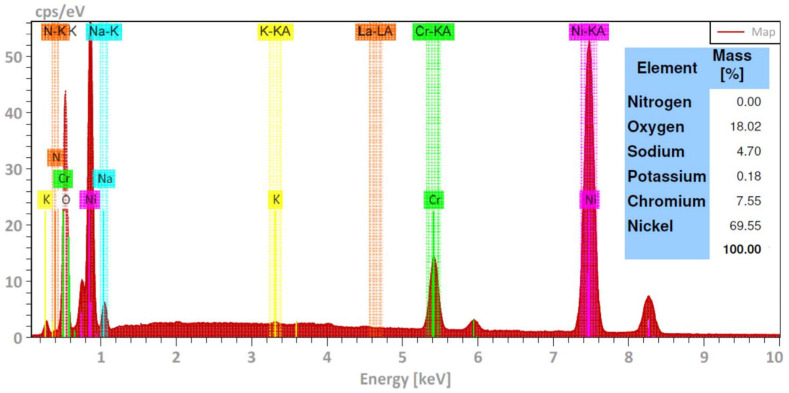
Elemental chemical composition of the Ni20Cr alloy surface immersed in Solar Salt at 500 °C and 1000 h of immersion.

Figure 2 shows the surface appearance of the Ni20Cr alloy after 1000 h of immersion in the Solar Salt at the different test temperatures. According to the micrographs, at 300 °C, the significant presence of surface attack or corrosion products was not observed. At 400 °C, the micrographs also did not show a significant surface attack, as the marks of the surface finish before the corrosion test were still visible, but the presence of a thin layer of corrosion products was evident. At 500 °C, a similar appearance was observed to that observed at 400 °C; however, the presence of corrosion products was more noticeable. According to the elemental chemical composition of the alloy surface exposed to 500 °C (Figure 3), the corrosion products were mainly formed by oxides of the alloying elements and their possible association with Na. The morphological aspects observed were consistent with the mass gain measurements, that is, when the test temperature increased, the presence of corrosion products was greater. However, despite the increase in mass gain and, therefore, corrosion, this was insignificant as the marks of the surface finish were still present. Similar observations have been reported for alloys with high corrosion resistance [3].

**Figure 4:**
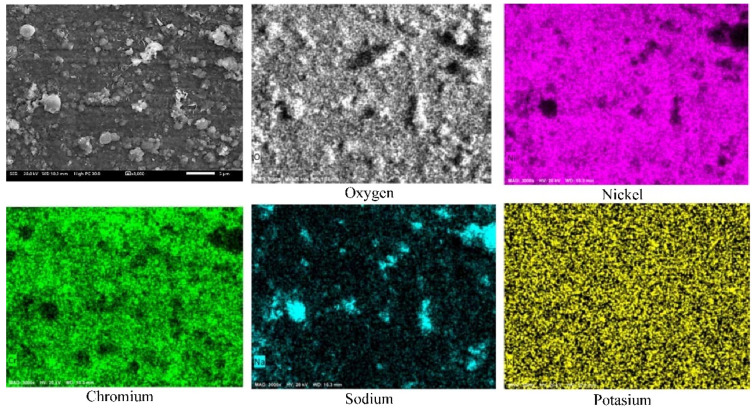
Element mapping of the Ni20Cr alloy surface immersed in Solar Salt at 500 °C and 1000 h of immersion.

Figure 4 shows the element mapping of the Ni20Cr alloy surface exposed to Solar Salt during 1000 h of immersion at 500 °C. According to the element mapping, it was observed that the corrosion products were mainly associated with elements of the alloy and oxygen, possibly Cr and Ni oxides. However, the presence of Na and K was also observed.

**Figure 5:**
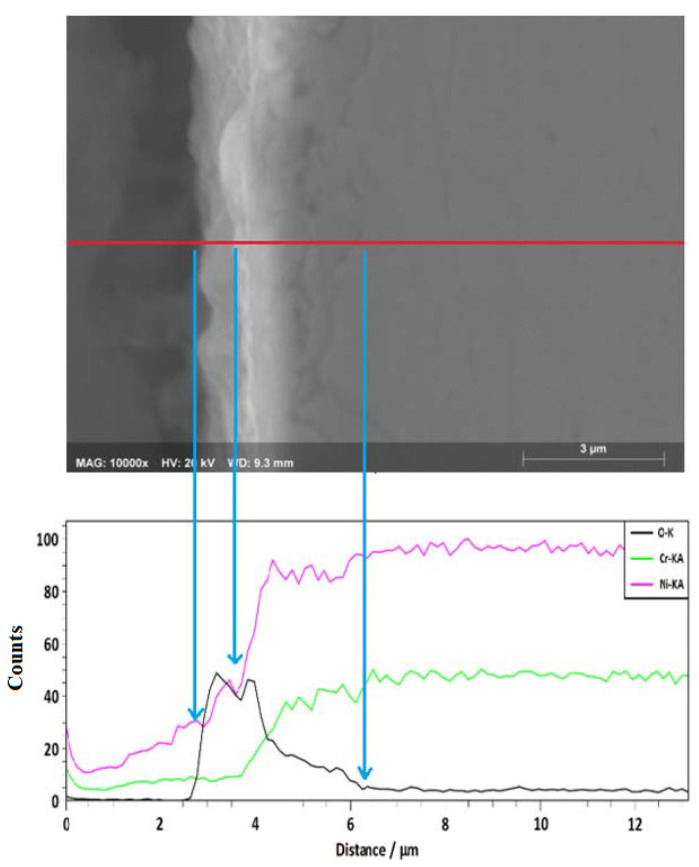
EDS line-scan of Ni20Cr alloy immersed in Solar Salt 500 °C for 1000 h.

A cross-sectional analysis (Figure 5) showed that a thin layer of corrosion products (<1 micron) was formed on the surface of the alloy, as well as possible internal damage with a depth close to 3 microns. The apparent internal damage observed may be a consequence of Cr or Ni depletion in the outer layers of the alloy due to the corrosion process [17]. However, an EDS line-scan showed the presence of oxygen outside and inside the alloy surface. This suggests the presence of a thin surface layer rich in Ni, Cr, and O (Cr and Ni oxides) and that the alloy underwent internal oxidation.

In the ternary salt (Figure 1), at 300 °C, the Ni20Cr alloy showed a mass gain in the first 250 h and subsequently a decrease until reaching an insignificant change in mass at the end of the test. The large mass changes observed up to 500 h may be associated with the initial formation of corrosion products strongly adhered to the alloy surface, but at longer immersion times, the corrosion process caused their detachment or partial dissolution. At 400 °C, a constant increase and decrease in mass was observed throughout the test. This may be due to a constant process of metallic dissolution and the formation of poorly adherent corrosion products. However, at 500 °C, a constant increase in the mass of the alloy was observed throughout the test, observing a mass gain greater than that obtained in the absence of La(NO_3_)_3_. It is possible that the presence of La(NO_3_)_3_ favored the corrosion process, or the presence of La^3+^ cations promoted the formation of a protective film. In all cases, the mass gain was greater than that observed in the absence of La(NO_3_)_3_. The mass gain detected at the highest test temperature is higher than those reported for other nickel-based alloys in binary or ternary metal nitrate salts [5]. This may be indicative of a more active corrosion process or the formation of protective layers due to the presence of La^3+^ cations.

**Figure 6:**
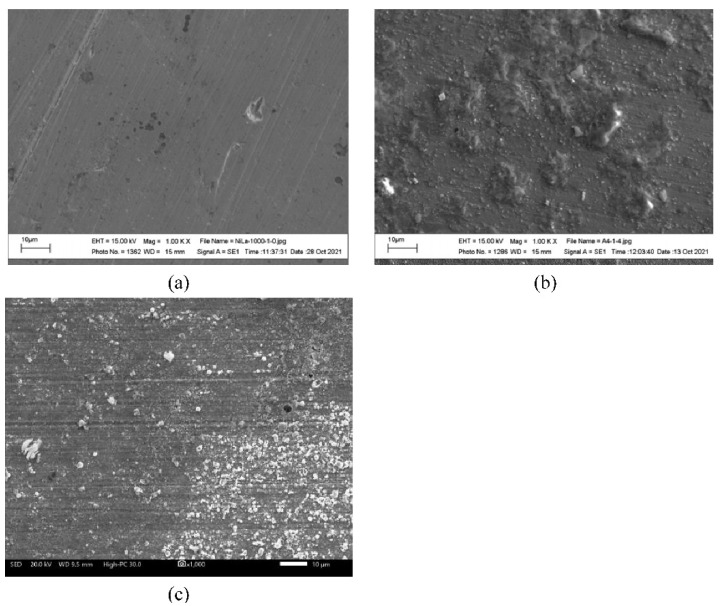
Morphological aspects of the Ni20Cr alloy surface immersed in the ternary salt after 1000 h and different test temperatures: (**a**) 300 °C, (**b**) 400 °C, and (**c**) 500 °C.

Figure 6 shows the surface appearance of the Ni20Cr alloy after 1000 h of immersion in the ternary salt at the different test temperatures. According to the micrographs, at 300 °C, only the isolated presence of corrosion products was observed without significant attack, and the presence of marks due to surface preparation was noticeable. At 400 °C, the presence of surface finish marks was still observed, and the formation of strongly adherent accumulations was evident, which may correspond to corrosion products or precipitates formed by the reduction of La^3+^ cations. At 500 °C, a higher density of corrosion products or precipitates was observed compared to that observed in the absence of La(NO_3_)_3_; however, it was still possible to observe the marks of the surface finish of the surface preparation process.

**Figure 7:**
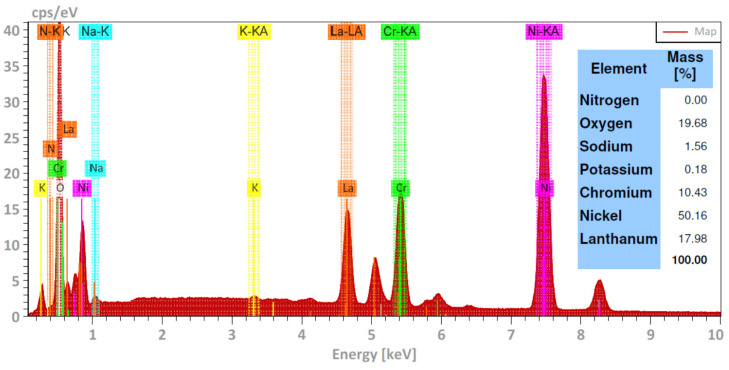
Elemental chemical composition of the Ni20Cr alloy surface exposed in the ternary salt at 500 °C and 1000 h of immersion.

Again, the morphological aspects observed were consistent with the mass gain measurements, that is, when the test temperature increased, the presence of corrosion products was greater due to the corrosion process; however, the higher density of corrosion products may be indicative of the greater metallic dissolution and/or the precipitation of protective layers due to the presence of La^3+^ cations. Elemental analysis of the alloy surface exposed to 500 °C (Figure 7) showed that the corrosion products were mainly formed by oxides of the alloying elements as well as possibly lanthanum oxide.

**Figure 8:**
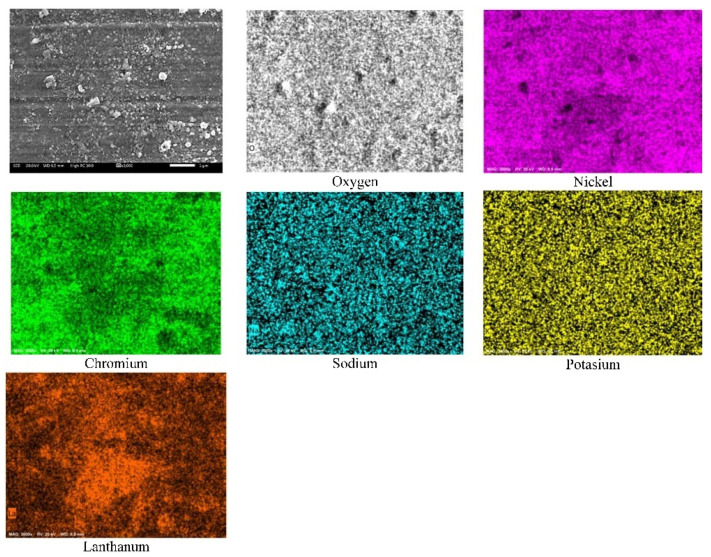
Element mapping of the Ni20Cr alloy surface immersed in the ternary salt at 500 °C and 1000 h of immersion.

Figure 8 shows the element mapping of the surface of the Ni20Cr alloy exposed to the ternary salt during 1000 h of immersion at 500 °C. According to the element mapping, in addition to the presence of the alloy elements and oxygen, possibly due to the presence of Ni and Cr oxides, a high density of lanthanum was also detected. Accordingly, the La^3+^ cations precipitated on the metal surface possibly as La oxides. The presence of Na and K was also detected; however, unlike what is observed in Figure 4, the distribution of Na was uniform. This suggests that the presence of La^3+^ cations limited the precipitation of Na-rich compounds.

**Figure 9:**
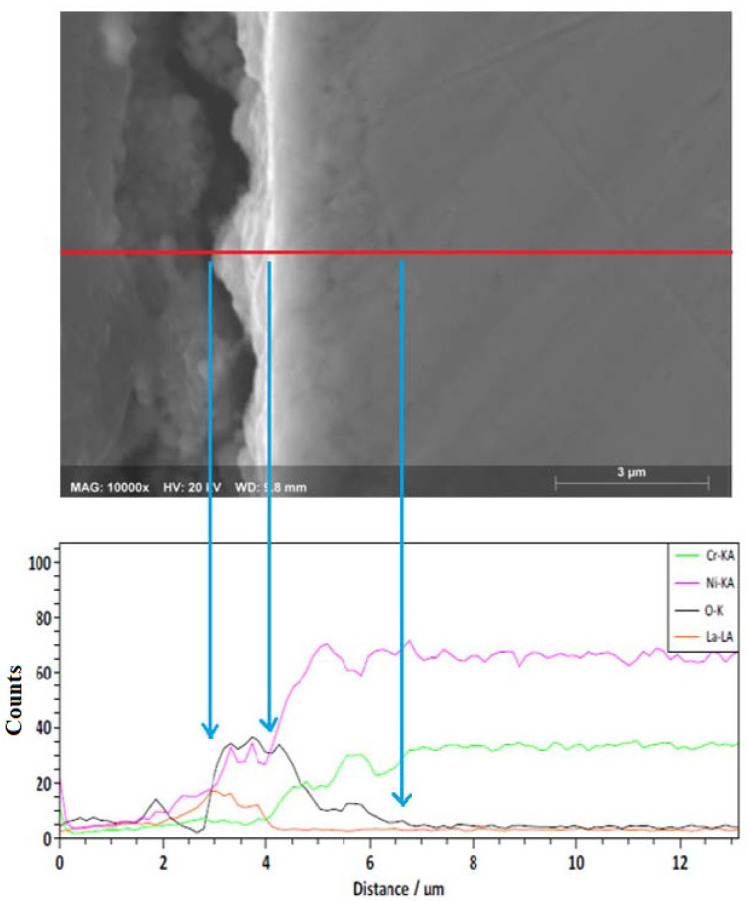
EDS line-scan of Ni20Cr alloy immersed in ternary salt 500 °C for 1000 h.

A cross-sectional analysis (Figure 9) showed that a layer of corrosion products rich in Ni-Cr-La-O was formed onto surface of the alloy, and its thickness was slightly greater than that formed in Solar Salt (Figure 5). Similarly, the EDS line-scan showed the presence of oxygen outside and inside the alloy surface. This corroborates the precipitation of La^3+^ cations on a thin layer of Cr and Ni oxides, as well as internal oxidation at a depth such as that observed in Solar Salt.

**Figure 10:**
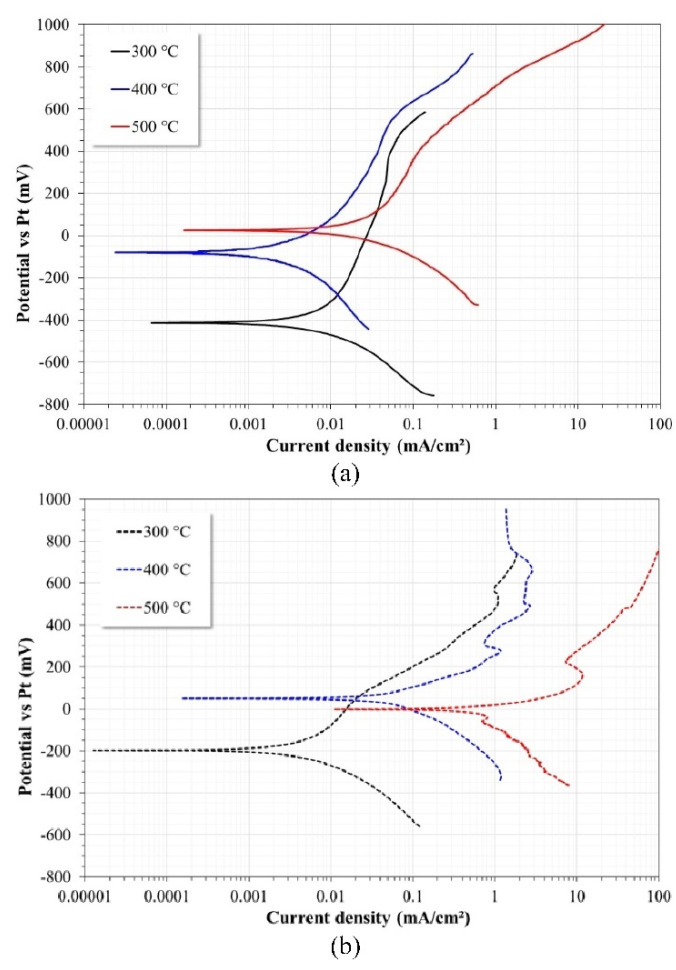
Potentiodynamic polarization curves of the Ni20Cr alloy immersed in: (**a**) Solar Salt and (**b**) ternary salt.

Additional tests were performed to confirm the effect of La(NO_3_)_3_. Figure 10 shows the polarization curves of the Ni20Cr alloy immersed in corrosive media at the different test temperatures. The electrochemical behavior of the Ni20Cr alloy in Solar Salt showed a displacement in the noble direction of the corrosion potential (Ecorr) with increasing test temperature. The cathodic branch showed a decrease in its current density from 300 °C to 400 °C and an increase when the temperature increased to 500 °C; this same behavior was observed in the values of the corrosion current density (Icorr). At all temperatures, the anodic branch showed an active behavior in a narrow range of potentials above the Ecorr value and, later, this behavior decreased to show a pseudo-passive behavior. However, the amplitude of the pseudo-passive behavior decreased with increasing temperature. Subsequently, at more active potentials again, an increase in current density was observed. Table 1 shows the electrochemical parameters obtained from the Tafel regions of the polarization curves. According to the tabulated values, it was observed that the anodic slope decreased with increasing temperature from 300 to 400 °C, and increased at 500 °C. The decrease in the anodic slope (from 300 to 400 °C) can be associated with the increase in fluidity and/or corrosiveness of the electrolyte, and the subsequent increase (from 400 to 500 °C) with the attempt of the alloy to form a protective layer. The cathodic slope showed a behavior opposite to the behavior observed in the anodic slope, that is, an increase when increasing the temperature from 300 to 400 °C, and subsequently a decrease when increasing the temperature from 400 to 500 °C. A linear relationship between the values of the Tafel slopes with the increase in temperature was not observed, as the electrochemical response is a function of both the behavior of the material and the corrosivity of the electrolyte. The polarization resistance (Rp) values were determined from the slope of the E-I relationship around ±10 mV of the corrosion potential. The Rp values showed the same trend observed in the Icorr values and the behavior of the anodic slope. According to the reported values, it was observed that at 300 °C and 400 °C, the corrosion rate (CR) did not show significant changes, but at 500 °C, it increased by an order of magnitude.

The corrosion rate values (mm/year) reported were determined from the Icorr values in accordance with the ASTM G102 standard (Standard Practice for calculation of Corrosion Rates and Related Information from Electrochemical Information):

(2) $$CR\;\left(\frac{mm}{year}\right)=K\;\frac{I_{corr}}{\rho \sum \frac{W_{i}}{n_{i}f_{i}}},$$

where *K* = 3.27 × 10^−3^ (mm⋅g/μA⋅cm⋅year), *I_corr_* = corrosion current density (μA/cm^2^), *EW* = equivalent weight of the material (dimensionless), *ρ* = density of the material (g/cm^3^), *f_i_* = mass fraction of component *i* of the alloy, *W_i_* = atomic weight, and *n_i_* = valence. 

In ternary salt, the behavior of the Ni20Cr alloy showed an increase in the corrosion potential (Ecorr) compared to that observed at 300 °C; however, at 400 °C and 500 °C, the Ecorr values were between +50 mV and −5 mV. The cathodic branch showed an increase in its current density with increasing temperature, as well as a clear increase in the values of the corrosion current density (Icorr). At 300 °C, the anode branch showed a behavior such as that observed in Solar Salt; however, the magnitude of the pseudo-passive zone was shorter, and at higher temperatures, only active behavior was observed. Table 2 shows the electrochemical parameters obtained from the Tafel regions. In this case, according to the tabulated values, it was observed that the anodic slope decreased with the increase in temperature, and the values of the cathodic slope increased with increasing temperature, with both behaviors being due to the increase in the corrosivity of the electrolyte and to the inability of the alloy to form a stable protective layer. This is consistent with the magnitude and trend of the reported Rp values. The corrosion rate values showed that it increased by an order of magnitude with each increase in temperature, and except for the data at 300 °C, at higher temperatures, the values were higher than those obtained in Solar Salt. The values of the electrochemical parameters were obtained directly from the polarization curves assuming the absence of ohmic drop.

According to the corrosion rate values obtained in Solar Salt, it was observed that the Ni20Cr alloy showed excellent corrosion resistance. Similar behavior has been observed for this alloy when evaluated as a coating in chloride-rich media (ZnCl_2_-KCl) at 450 °C; its Icorr values were around 4 mA/cm^2^ and 28 mA/cm^2^ when applied by combustion powder spray [14] and the HVOF process [15], respectively. Similarly, the alloy evaluated in vanadium salts (NaVO_3_) at 700 °C showed Icorr values of 0.5 mA/cm^2^ [16]. However, with the addition of La(NO_3_)_3_, it was observed that its presence caused an increase in the corrosivity of the Solar Salt. Notwithstanding this, it must be considered that this type of test only reflects the initial behavior of the alloy and that it can change at longer immersion times, either due to the development of protective layers that would reduce its rate of degradation or due to the inability of the alloy to self-passivate, which would increase its rate of degradation.

### 3.2. Reaction Mechanisms

**Figure 11:**
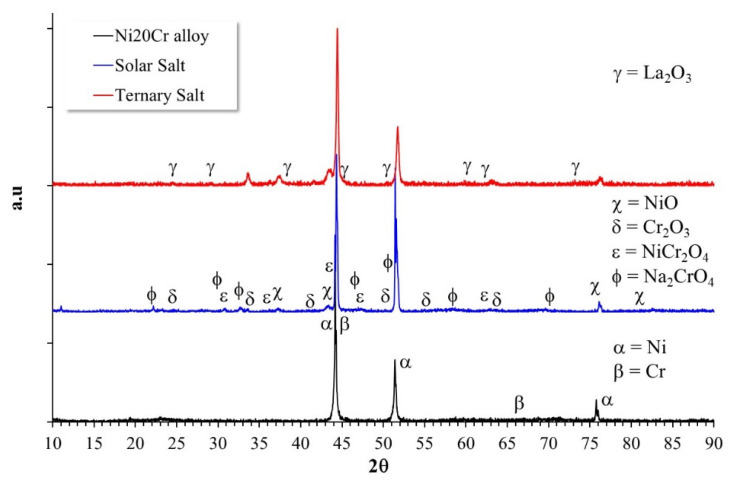
Diffractogram of the Ni20Cr alloy surface before and after the corrosion test on Solar Salt and ternary salt at 500 °C after 1000 h of immersion.

X-ray diffraction analysis of the Ni20Cr alloy surface before and after the corrosion tests (Figure 11) showed that the alloy before the corrosion test showed a main peak at 2θ = 44.21°. It has been reported [18] that this signal is due to the superposition of the peaks corresponding to Ni (1 1 1) 2θ = 44.505° and Cr (1 1 1) 2θ = 44.390°, which suggests the formation of a Ni-Cr solid solution. Similar diffractograms have been reported in other studies for Ni-Cr alloys [16,19,20]. The diffractograms of the corroded surfaces did not show significant changes with respect to that observed for the noncorroded alloy. This is due to the low mass gain observed, which suggests the formation of a thin layer of corrosion products. However, in both corrosive media, it was possible to detect the presence of Cr and Ni oxides (Cr_2_O_3_, NiO) and Ni chromate (NiCr_2_O_4_), which is considered a protective corrosion product [5,7], as well as the additional presence of the oxide of La (La_2_O_3_) on the surface of the corroded alloy in the ternary salt. The presence of this compound indicates that the La^3+^ cations favored the formation of a protective layer on the surface of the alloy. This shows that the highest mass gain observed in the ternary salt (Figure 1) is also associated with this process.

Based on the EDS and XRD analysis, as well as the gravimetric and electrochemical behavior of the Ni20Cr alloy, it is possible to determine the molten salt–alloy interaction that led to the observed surface changes. In general, the corrosion mechanism depends mainly on the type of anion in the molten salt, where those based on nitrates correspond to oxyanionic salts and the concentration of the oxide ion (O^−2^) defines the basicity of the melt. The importance of the basicity of the melt on the dissolution mechanism of the metallic surface and its protective layers has already been previously documented [21,22,23,24,25,26,27]. In the case of Solar Salt, in the molten state, it experiences the following decomposition reactions, and its reaction rate increases with increasing temperature [1,12,28,29,30]:

(3) $${\mathrm{NO}}_{3}^{-}↔{\mathrm{NO}}_{2}^{-}+\frac{1}{2}O_{2},$$

The nitrite ion (NO^−2^) formed after decomposition can undergo secondary decomposition reactions according to [1,12,28,29,30]:

(4) $$2{\mathrm{NO}}_{2}^{-}↔O^{2-}+\mathrm{NO}+{\mathrm{NO}}_{2}\;,$$

(5) $$5{\mathrm{NO}}_{2}^{-}↔O^{2-}+{\;N}_{2}+3{\mathrm{NO}}_{3}^{-},$$

Then, in the presence of these ionic species, it is possible that the formation of the metal oxides detected occurred by the surface reaction of the alloy with the oxygen generated by reaction (3) according to [30]:

(6) $$2\mathrm{Cr}+\frac{3}{2}O_{2}↔{\mathrm{Cr}}_{2}O_{3},$$

(7) $$\mathrm{Ni}+\frac{1}{2}O_{2}↔\mathrm{NiO},$$

The above reactions favor the further decomposition of the nitrate ion to nitrite ion, due to the consumption of the O_2_ that drives the reaction forward. In general, the above reactions can be simplified to (where R is Na or K) [2]:

(8) $$2{\mathrm{RNO}}_{3}+2\mathrm{Cr}↔{\mathrm{Cr}}_{2}O_{3}+2\mathrm{NO}+R_{2}O,$$

(9) $$2{\mathrm{RNO}}_{3}+3\mathrm{Ni}↔3\mathrm{NiO}+2\mathrm{NO}+R_{2}O,$$

The formation of Na and K oxides (O^−2^ ions) increases the basicity of the melt [21,22], causing a process of basic dissolution of the protective oxides, thereby favoring the formation of metallic chromates [24]:

(10) $$\mathrm{NiO}+{\mathrm{Cr}}_{2}O_{3}\overset{}{↔}{\mathrm{NiCr}}_{2}O_{4},$$

All these compounds were detected on the metal surface as corroborated by EDS and XRD analyses. On the other hand, EDS analyses indicated the presence of deposits with Na and K, whose formation may be due to the following reactions [31,32]:

(11) $${\mathrm{Cr}}_{2}O_{3}+4{\mathrm{NaNO}}_{3}\to 2{\mathrm{Na}}_{2}{\mathrm{CrO}}_{4}+3{\mathrm{NO}}_{2}+\mathrm{NO},$$

(12) $${\mathrm{Cr}}_{2}O_{3}+4{\mathrm{KNO}}_{3}\to 2K_{2}{\mathrm{CrO}}_{4}+3{\mathrm{NO}}_{2}+\mathrm{NO},$$

(13) $${\mathrm{Cr}}_{2}O_{3}+6{\mathrm{NaNO}}_{2}\to 2{\mathrm{Na}}_{2}{\mathrm{CrO}}_{4}+6\mathrm{NO}+{\mathrm{Na}}_{2}O,$$

These reactions are favored by the basic character of the molten salt (high concentration of oxide ion, O^2−^) whose reaction product is soluble in the melt and can precipitate as a porous layer on the surface of the alloy [33]. The formation of Na_2_CrO_4_ and K_2_CrO_4_ is favored with increasing temperature; therefore, the corrosion resistance of the alloy could be diminished as its formation depletes the Cr content of the protective layer [33,34]. Recent studies [12] have shown that Solar Salt dissolves pure Cr by similar reactions.

In the case of the ternary salt, in addition to the above reactions, the decomposition of lanthanum nitrate can occur according to the reaction proposed for metal nitrate salts [5,8]:

(14) $$\mathrm{La}{\left({\mathrm{NO}}_{3}\right)}_{3}\overset{}{↔}\mathrm{La}{\left({\mathrm{NO}}_{2}\right)}_{3}+1\frac{1}{2}O_{2},$$

(15) $$2\mathrm{La}{\left({\mathrm{NO}}_{2}\right)}_{3}\overset{}{↔}{\mathrm{La}}_{2}O_{3}+3\mathrm{NO}+3{\mathrm{NO}}_{2},$$

The above reaction justifies the deposition of lanthanum oxide on the surface of the alloy as reported in the previous sections. In addition, due to the higher molecular weight of La, with respect to any other metallic cation present, its deposition would cause a greater mass gain than that observed in Solar Salt. However, in addition to the above, the decomposition of one mole of La(NO_3_)_3_ would triple the concentration of oxidizing species than those generated by the decomposition of one mole of NaNO_3_ or KNO_3_ (reactions 3 to 5). This would increase the oxidizing power and basicity of the melt.

**Figure 12:**
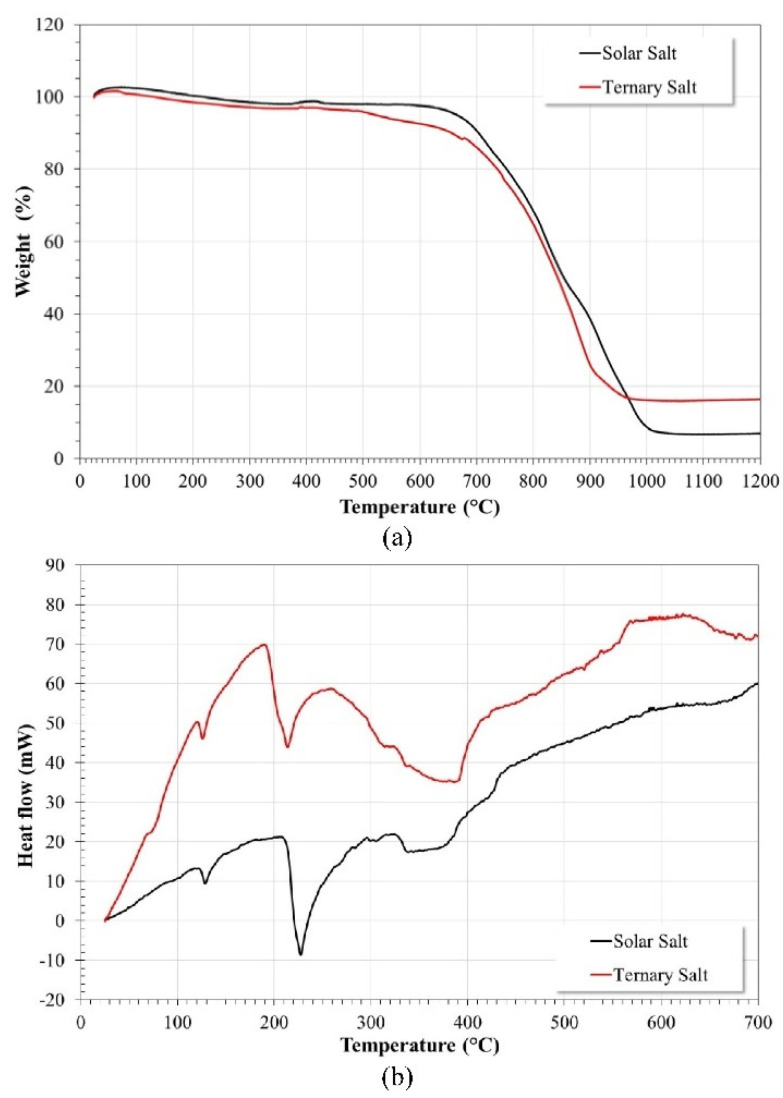
TGA-DSC Analysis of Solar Salt and ternary salt. (**a**) TGA curves, (**b**) DSC curves.

To determine the changes that the addition of La(NO_3_)_3_ causes to the Solar Salt, TGA-DSC (STA 449 F1 Jupiter) analyses were performed (Figure 12). According to the TGA diagram, in the temperature range of the corrosion tests carried out, the ternary salt experienced a greater mass loss than the Solar Salt did. This may be associated with the low melting point of La(NO_3_)_3_ (69.9 °C) and the higher number of species generated by its dissociation (reactions 3 to 5). In addition, the thermal stability of Solar Salt is approximately up to 600 °C [7], and, with the addition of La(NO_3_)_3_, it is reduced to approximately 500 °C, which is the temperature from which the onset of the greatest mass loss is observed. 

It has been reported that the decomposition of metal nitrate salts proceeds according to the following steps [35,36,37]: the first step involves the formation of nitrites with the release of oxygen according to reaction 3; the second step is a secondary decomposition process of nitrite releasing nitrogen or nitrogen oxides according to reactions 4 and 5, and simultaneously forming metallic oxides in a similar way to that indicated in reaction 15. In the case of Solar Salt, the first stage of thermal decomposition involved a loss of mass of around 5% up to 650 °C, and from that temperature, the second stage of thermal decomposition was observed until reaching a mass loss of the order of 93%. On the other hand, in the ternary salt, the first stage of thermal decomposition involved a mass loss of around 11% up to 675 °C, followed by the second stage of thermal decomposition until reaching a mass loss of around 84%.

According to the DSC spectrum, the melting point of the ternary salt was lower than that of the Solar Salt (13.51 °C lower), and the start of melting was also reduced (17.84 °C lower). These changes can be positive as they increase the range of fluidity of the ternary salt [7]. However, the reduction in thermal stability causes an increase in the concentration of oxidant species, basicity, and fluidity of the salt, which induces greater aggressiveness. It is possible that the addition of lower concentrations of La(NO_3_)_3_ may reduce the aforementioned effects and a beneficial effect of La^3+^ cations may be observed.

## 4. Conclusions

Based on the results derived from this study, it was observed that:

(1) The addition of La(NO_3_)_3_ into Solar Salt, at the concentration evaluated here, decreased its thermal stability and increased its corrosivity.
(2) The observed changes showed a decrease of 13.51 °C in the melting point, and a decrease of 100 °C in the beginning of the decomposition point of Solar Salt.
(3) These changes caused an increase in the concentration of oxidizing species, fluidity of the ternary salt, as well as its basicity.
(4) The presence of the (La^3+^) formed a protective layer on the surface of the alloy.
(5) The Ni20Cr alloy, immersed in the Solar Salt, developed protective corrosion products such as Cr and Ni oxides (Cr_2_O_3_, NiO) and Ni chromate (NiCr_2_O_4_), and when immersed in the ternary salt, the additional presence of a La-based protective layer (La_2_O_3_) was detected.

## Figures and Tables

**Table 1:** Electrochemical parameters of Ni20Cr alloy immersed in Solar Salt at different temperatures.

Temperature	Ecorr	Icorr	βa	βc	CR	Rp
	(mV)	(μA/cm^2^)	(mV/Decade)	(mV/Decade)	(mm/Year)	(Ohm-cm^2^)
300 °C	−414 ± 7	9.50 ± 1	897 ± 15	−272 ± 15	0.093 ± 0.005	7300 ± 100
400 °C	−80 ± 6	3.60 ± 2	405 ± 10	−392 ± 8	0.035 ± 0.006	17,000 ± 150
500 °C	23 ± 3	30.3 ± 3	626 ± 7	−236 ± 10	0.298 ± 0.010	1870 ± 50

**Table 2:** Electrochemical parameters of Ni20Cr alloy immersed in ternary salt at different temperatures.

Temperature	Ecorr	Icorr	βa	βc	CR	Rp
	(mV)	(μA/cm^2^)	(mV/Decade)	(mV/Decade)	(mm/Year)	(Ohm-cm^2^)
300 °C	−195 ± 6	4.80 ± 0.5	380 ± 15	−211 ± 10	0.048 ± 0.003	9500 ± 200
400 °C	49 ± 3	75.60 ± 3	151 ± 7	−249 ± 15	0.745 ± 0.05	500 ± 50
500 °C	−3 ± 2	672.60 ± 10	92 ± 6	−359 ± 20	6.626 ± 0.50	30 ± 7

## Data Availability

All data are available upon request through contacting the corresponding author at jporcayoc@gmail.com.
